# The risks of hormones to the inner ear

**DOI:** 10.1016/j.bjorl.2026.101823

**Published:** 2026-04-30

**Authors:** Lucas Resende Lucinda Mangia, Raquel Mezzalira, Roseli Saraiva Moreira Bittar

**Affiliations:** a Universidade Federal do Paraná (UFPR), Department of Otolaryngology, Curitiba, PR, Brazil; b Universidade Estadual de Campinas (UNICAMP), Department of Otolaryngology, Campinas, SP, Brazil; c Faculdade de Medicina da Universidade de São Paulo (FMUSP), Department of Otolaryngology, São Paulo, SP, Brazil

Sex hormones have long been recognized as central regulators of reproductive physiology, metabolism, vascular homeostasis, and neural excitability. Yet their influence on auditory and vestibular function remains poorly explored in routine otolaryngological practice. Growing scientific evidence indicates that estrogen, progesterone, and androgens exert direct and indirect effects on cochlear and vestibular physiology. As hormonal therapies become increasingly common in both medical and non-medical settings, clinicians are encountering new situations in which inner-ear symptoms may have a hormonal component, a possibility that often remains unrecognized.

The inner ear is not a hormonally neutral tissue, as demonstrated by seminal basic science studies.^1^ Sex steroid receptors have been identified in cochlear and vestibular structures, including hair cells, spiral ganglion neurons, the stria vascularis, and vestibular sensory epithelia. Estrogen appears to contribute to inner-ear homeostasis through multiple mechanisms, including regulation of labyrinthine blood flow, maintenance of endolymphatic ionic gradients, and neuroprotective effects on auditory and vestibular pathways. Progesterone may also influence neuronal excitability and fluid regulation within the labyrinth. These mechanisms help explain why endocrine transitions frequently coincide with fluctuating auditory and vestibular symptoms.^2^

Although less extensively studied than estrogen, androgens may also influence inner-ear function through multiple pathways. They can alter vascular tone, blood viscosity, and endothelial function, potentially predisposing susceptible individuals to transient disruptions in local perfusion. In addition, androgens may promote systemic metabolic changes, including dyslipidemia, increased hematocrit, and insulin resistance, that further aggravate these disturbances. At the central level, they may modulate neuronal responsiveness through effects on GABAergic and glutamatergic neurotransmission, with potential repercussions on tinnitus perception, auditory sensitivity, and vestibular tolerance.

Moreover, clinical studies support the influence of hormonal fluctuations on inner-ear function. Many women report worsening tinnitus, episodic vertigo, auditory discomfort, or aural fullness during specific phases of the menstrual cycle, pregnancy, the postpartum period, and the menopausal transition. Likewise, some patients experience dizziness or sudden changes in tinnitus intensity shortly after the initiation, withdrawal, or dose adjustment of hormonal medications. Observations from specific clinical disorders further reinforce this association, as several labyrinthine conditions appear to be modulated by sex hormones.^2^ Vestibular Migraine provides one of the clearest examples: abrupt estrogen decline may trigger trigeminovascular activation and intensify vestibular and auditory symptoms. Not surprisingly, middle-aged women frequently present with auditory and vestibular complaints during the perimenopausal years, often in clinical contexts attributable to vestibular migraine. Androgenic fluctuations may also indirectly influence this condition by altering the estrogen–progesterone balance and thereby modulating trigeminovascular excitability.

Hormonal influences may also underlie clinical presentations that resemble Menière's disease, since hormonal disturbances can induce pathophysiological changes that plausibly account for these manifestations while also intersecting with mechanisms implicated in endolymphatic dysfunction. Moreover, in patients with Ménière’s disease itself, the hormonal milieu has been shown to influence both symptom expression and disease course.^3^

These considerations become particularly relevant in the context of the expanding use of hormonal interventions beyond established endocrine indications. Hormone replacement therapy, compounded formulations, subcutaneous hormonal implants, testosterone supplementation, and so-called metabolic or anti-aging hormonal protocols have become increasingly common across diverse clinical and non-clinical settings. In Brazil, this debate gained particular relevance after the Brazilian Health Regulatory Agency (Agência Nacional de Vigilância Sanitária - Anvisa) suspended, in October 2024, the commercialization, advertising, and use of manipulated hormonal implants popularly known as “beauty chips.”^4^ These formulations frequently contained combinations of gestrinone, testosterone, oxandrolone, and other hormonal agents delivered through implantable preparations lacking robust evidence of safety, efficacy, and dose standardization for aesthetic purposes, fatigue, non-specific menopausal symptoms, or performance enhancement. The regulatory decision followed reports from medical societies describing a growing number of patients presenting with adverse systemic effects, including dyslipidemia, hypertension, arrhythmias, thromboembolic events, insomnia, affective symptoms, voice changes, and androgenic manifestations.^5^ Apart from the direct hormonal impact on the inner ear, several of these systemic alterations may promote pathophysiological mechanisms involved in auditory and vestibular dysfunction.

While current safety discussions surrounding these interventions have focused predominantly on cardiovascular, endocrine, and metabolic consequences, potential neurotological repercussions remain largely absent from clinical surveillance. For the otolaryngologist, this represents an important diagnostic blind spot. Patients presenting with tinnitus, episodic dizziness, unexplained aural fullness, sound sensitivity, fluctuating auditory symptoms, or poorly characterized vestibular complaints may not spontaneously report hormonal therapies unless actively questioned. In many cases, the temporal relationship between symptom onset and endocrine intervention remains unrecognized, allowing hormonal contributors to go overlooked.

A detailed hormonal history should therefore become part of routine neurotological assessment. Menopausal status, contraceptive use, fertility treatments, androgen supplementation, and recent exposure to hormonal therapies should be deliberately explored, particularly when symptoms fluctuate without clear audiological correlation or when conventional vestibular diagnoses fail to fully explain the clinical picture. Hormonal influences should also be suspected in audiovestibular disorders showing an unexpectedly unfavorable course, recurrent exacerbations, or poor response to conventional therapeutic measures. In patients with established audiovestibular disorders, hormonal transition periods and exposure to exogenous sex hormones should always be considered potential contributors to clinical instability. Accordingly, physicians involved in their care ought to incorporate these aspects into patient counseling, therapeutic planning, and prognostic assessment.

## ORCID IDs

Lucas Resende Lucinda Mangia: 0000-0003-3443-3640

Raquel Mezzalira: 0000-0002-0279-8007

Roseli Saraiva Moreira Bittar: 0000-0001-8731-8908

## Funding

There is no financial or material support.

## Data availability statement

N/A.

## Declaration of competing interest

The authors declare no conflicts of interest.
